# EjMYB15 Improves Cold Tolerance of Postharvest Loquat Fruit via Upregulating Antioxidant Enzyme Genes

**DOI:** 10.3390/foods15020301

**Published:** 2026-01-14

**Authors:** Weiqi Liang, Jiahui Wan, Jing Lin, Yanting Wu, Wenbing Su, Zhongqi Fan

**Affiliations:** 1 Institute of Postharvest Technology of Agricultural Products, College of Food Science, Fujian Agriculture and Forestry University, Fuzhou 350002, China; 18535361607@163.com (W.L.); jhuiwan@163.com (J.W.); 15396028003@163.com (J.L.); wuyanting2024@163.com (Y.W.); 2 Fruit Research Institute, Fujian Academy of Agricultural Science, Fuzhou 350013, China

**Keywords:** loquat, chilling injury, melatonin, MYB transcription factor, ROS scavenging

## Abstract

As cold-sensitive fruits, loquats easily develop chilling injury (CI) during cold storage, which leads to quality deterioration and economic losses. Our prior research indicated that exogenous melatonin (MT) treatment can mitigate CI in postharvest loquats by regulating reactive oxygen species (ROS) metabolism, but the underlying molecular mechanism remains unclear. The primary objective of this study is to decipher the molecular regulatory pathway by which MT alleviates CI in postharvest loquats, focusing on the role of MYB transcription factors (TFs) in modulating antioxidant enzyme genes. Here, MT treatment remarkably reduced CI severity in loquat fruits, as reflected by lower CI index, reduced cell membrane permeability, decreased firmness, lower a* and b* values, and higher L* value, compared with the control group. Moreover, a cold-induced MYB TF, designated EjMYB15, was identified. Compared to non-treated fruits, the expression level of *EjMYB15* was maintained at higher levels in MT-treated loquats. Subcellular localization and transactivation assays demonstrated that EjMYB15 is a nuclear-localized transcriptional activator. Electrophoretic mobility shift assay (EMSA) and dual-luciferase reporter (DLR) assays showed that EjMYB15 binds the MYB-binding sites (MBS) in the promoters of four antioxidant enzyme genes (*EjCAT1*, *EjCAT2*, *EjGST1*, and *EjGST2*), thereby activating their transcription. Taken together, these findings indicate that EjMYB15 positively regulates cold tolerance of loquat fruits by improving ROS scavenging capacity. These results elucidate the regulatory pathway by which MYB TFs mitigate CI and provide new theoretical support for the application of MT in alleviating CI in postharvest fruits.

## 1. Introduction

Loquat (*Eriobotrya japonica* Lindl.) is highly favored among consumers due to its rich nutritional composition, including carbohydrates, vitamins, minerals, organic acids, and various bioactive compounds [1]. However, postharvest loquat fruits exhibit vigorous physiological metabolism, making them highly susceptible to spoilage and thus significantly shortening their shelf life [2,3]. Low-temperature storage is widely recognized as a critical approach to preserving postharvest fruit quality and extending storage duration [3]. Unfortunately, loquat fruits are vulnerable to chilling injury (CI) under cold stress, characterized by typical symptoms including browning, pitting, and flesh lignification. These adverse effects ultimately lead to quality deterioration and substantial economic losses in the loquat industry [4,5,6]. Therefore, developing efficient strategies to improve cold tolerance is crucial for the postharvest handling and transportation of loquat fruits.

Melatonin (N-acetyl-5-methoxytryptamine, MT), a naturally occurring indoleamine compound, is known as a pivotal regulator in preserving the postharvest quality of fresh products [7]. It not only effectively delays fruit senescence and preserves postharvest quality but also enhances the cold tolerance of horticultural crops to alleviate CI, thereby extending shelf life and improving commercial value [8]. For instance, MT treatment stabilizes the cell membrane of peach fruits by inhibiting membrane lipid peroxidation and keeping a high ratio of unsaturated to saturated fatty acids [9]. In tomatoes, MT alleviates CI development during low-temperature storage by increasing the activities of energy metabolism-related enzymes to sustain adenosine triphosphate (ATP) levels [10]. In ‘Nanguo’ pears, MT application mitigates peel browning by modulating phenolic compounds and proline metabolism [11]. In bananas, MT reduces CI incidence through the modulation of reactive oxygen species (ROS) metabolism [12]. Our previous research has shown that MT can effectively alleviate CI of loquat fruits via improving ROS scavenging ability; however, the underlying molecular regulatory mechanism remains elusive.

Cold stress typically induces excessive ROS accumulation in plant cells, triggering a cascade of oxidative damage events that disrupt cellular homeostasis [13]. To prevent such oxidative stress caused by ROS overaccumulation, plants can develop an intrinsic antioxidant defense system that serves to scavenge excess ROS and keep cellular redox balance. This antioxidant system comprises enzymatic and non-enzymatic components. Enzymatic antioxidants primarily include catalase (CAT), peroxidase (POD), superoxide dismutase (SOD), ascorbate peroxidase (APX), dehydroascorbate reductase (DHAR), monodehydroascorbate reductase (MDHAR), glutathione reductase (GR), and glutathione S-transferase (GST) [14,15,16]. Accumulating evidence has demonstrated that enhancing the ROS scavenging capacity in the antioxidant system is important for enhancing cold tolerance of horticultural crops. For example, fucoidan treatment enhances peach fruit cold tolerance by upregulating the activities and gene expression levels of antioxidant enzymes (CAT, POD, APX, SOD, MDHAR, GR, and DHAR) [17]. Similarly, L-glutamic acid (L-Glu) mitigates cold damage in prunes by upregulating the activities of CAT, APX, and SOD, thereby increasing ROS scavenging ability and alleviating oxidative stress during cold storage [18]. In line with these observations, our previous work indicated that MT treatment alleviates CI in loquats, which is attributed to increased contents of non-enzymatic components, including ascorbic acid (AsA) and glutathione (GSH), and enhanced activities of CAT, APX, and SOD [8].

The MYB transcription factor (TF) family represents one of the most expansive TF families in plants, with pivotal functions in modulating plant growth, development, secondary metabolism, hormone signaling transduction, and tolerance to biotic and abiotic stresses. MYB TFs exert their regulatory functions by binding to specific *cis*-acting elements (MYB-binding site, MBS) in their promoters of target genes, thereby participating in various stress response pathways. Mounting evidence has indicated that multiple MYB members are involved in the regulation of plant cold stress responses. For instance, PuMYB21 and PuMYB54 in ‘Nanguo’ pears target the promoter of *PuPLDβ1* (a crucial enzyme catalyzing membrane phospholipid hydrolysis) and promote its expression, thereby accelerating membrane phospholipid degradation and ultimately leading to peel browning during cold storage [19]. In bananas, MaMYB13 participates in cold stress response via activating the expression of membrane lipid metabolism-related genes [20]. Peanut AhMYB30 improves resistance to freezing stresses in Arabidopsis by modulating both the DREB/CBF and ABA signaling pathways [21]. In tomatoes, SlMYB13 promotes cold tolerance through SlMYC2-mediated jasmonic acid signaling that targets SlHSP17.7J [22]. In the present study, we demonstrate that EjMYB15 mediates melatonin (MT)-induced cold tolerance in postharvest loquat fruits by transcriptionally activating antioxidant enzyme genes to enhance ROS scavenging capacity, laying a theoretical foundation for developing CI mitigation strategies in the postharvest loquat industry.

## 2. Materials and Methods

### 2.1. Fruit Material and Treatments

Loquat fruits (*E. japonica* cv. Guifei) were harvested 142 days after flowering at the National Germplasm Resource Nursery for Longan and Loquat, Fujian Academy of Agricultural Sciences. Healthy loquats of uniform size and color, and devoid of disease, pest infestation, and mechanical injury, were chosen for the experiment. The MT-treated loquats were soaked in 50 μmol L^−1^ melatonin solution for 10 min, and the control loquats were immersed in distilled water for the same duration. After treatment, all loquats were stored at a temperature of (4 ± 1) °C with a relative humidity of 90% for a duration of 20 days. We sampled loquat pulp tissues on days 0, 4, 8, 12, 16, and 20 and stored them at −80 °C for further experiment [8].

### 2.2. Physiological Indexes

The progression of CI symptoms in loquats was monitored continuously and documented photographically at specific storage days (0, 4, 8, 12, 16, and 20 days). The CI levels were categorized as follows: 0 (no visible symptoms); 1 (slight, browning area < 5%); 2 (moderate, browning area 5–25%); 3 (severe, browning area 25–50%); and 4 (very severe, browning area > 50%). CI index = Σ (CI scale × number of fruits in each scale)/(total number of fruits) [23,24].

Relative electrolyte leakage (%) was detected using the method of Rui et al. [25]. Twenty loquat pulp disks (0.5 cm in diameter each) from 10 fruits were submerged in 25 mL distilled water. After 3 h, the initial solution conductivity (L_0_) was measured with a desktop conductivity meter (model SG68, Mettler-Toledo, Greifensee, Switzerland). The solution was then boiled for 30 min, adjusted back to a volume of 25 mL, and the total conductivity (L_1_) was recorded. The relative electrolyte leakage = (*L*_0_/*L*_1_) × 100 and is expressed as %.

According to the method of Yang et al. [26], fruit firmness was assessed by a texture analyzer (TMS-PIOT, Food Technology Corporation, Sterling, VA, USA) with a test speed of 200 mm min^−1^, and the results were expressed in N.

For fruit appearance determination, 10 loquat fruits were randomly selected to evaluate the fruit appearance lightness (L*), chromaticity a*, and b* using a chroma meter (CS-580A, Hangzhou CHNSpec Technology Co., Ltd., Hangzhou, China) [27].

### 2.3. RNA Extraction, Gene Isolation, and Sequence Analysis

Following the manufacturer’s protocol for Tiangen Biotech RNAprep Pure Plant Kit (Tiangen, Beijing, China), total RNA was extracted from loquat pulp. The first-strand cDNA synthesis was performed using the cDNA synthesis mix kit (Lablead, Beijing, China). Quantitative real-time PCR (RT-qPCR) was conducted using the SYBR qPCR mix kit (Vazyme, Nanjing, China) and carried out using a CFX96 Real-Time PCR System machine (Bio-Rad, Hercules, CA, USA).

RT-qPCR Experimental Steps:

(a) Prepare a 10 μL RT-qPCR reaction system with the following components:
  5 μL of ChamQ Blue Universal SYBR qPCR Master Mix,
  1 μL of cDNA template,
  0.2 μL of each primer,
  3.6 μL of nuclease-free water.

(b) Set the RT-qPCR reaction program as follows:
  Initial denaturation: 95 °C for 30 s,
  Amplification stage: 40 cycles of 95 °C for 5 s and 60 °C for 30 s,
  Melt curve stage: 95 °C for 15 s, 60 °C for 1 min, followed by a gradual increase to 95 °C for 30 s with continuous fluorescence acquisition.

*EjACT* was selected as an endogenous reference gene [28], and all primers used in this study are listed in Table S1. The relative gene expression was calculated using the 2^−ΔΔCt^ method [29].

The coding sequence (CDS) of EjMYB15 (*Ej00040204*) was obtained from the loquat genome data (https://db.cngb.org/data_resources/project/CNP0001531/, accessed on 15 June 2023).

PCR Experimental Steps:

(a) Prepare a 50 μL PCR reaction system comprising the following components:
  25 μL of 2× Phanta Flash Master Mix (DyePlus),
  4 μL of cDNA template,
  3 μL of each primer,
  15 μL of nuclease-free water.

(b) Set the PCR reaction program as follows:
  Initial denaturation: 95 °C for 3 min,
  Amplification stage: 35 cycles of denaturation: 95 °C for 30 s,
  Annealing: 60 °C for 30 s,
  Extension: 72 °C for 2 min,
  Final extension: 72 °C for 10 min.

A phylogenetic tree of the protein sequences of EjMYB15 was constructed in MEGA7 software. The Neighbor-Joining (NJ) method was employed with the following parameters: the Kimura 2-parameter model was selected as the nucleotide substitution model, and the bootstrap method was performed with 1000 replicates to evaluate the reliability of the tree topology. We performed multiple sequence alignment using DNAMAN 10 software.

### 2.4. Subcellular Localization

The CDS of *EjMYB15* was cloned into the pBE-GFP vector to generate an EjMYB15-GFP fusion protein. The NLS-mCherry nuclear marker enabled visualization of subcellular localization. The empty pBE-GFP, EjMYB15-pBE-GFP, and NLS-mCherry were co-transfected into tobacco leaves for transient expression, respectively. After 48–72 h of post-infiltration, GFP and mCherry fluorescence were observed using an inverted fluorescent microscope (Zeiss, Oberkochen, Germany) [30].

### 2.5. Transcriptional Activation Assay in Yeast Cells

The CDS of *EjMYB15* was amplified and inserted into the pGBKT7 vector. The reconstructed plasmid pGBKT7-EjMYB15, along with pGBKT7-p53+pGADT7 (positive control) and pGBKT7-empty (negative control), was transformed into the Y2H yeast strain, respectively. The growth ability and X-α-galactosidase activity of yeast cells cultured on SD/-Trp-His-Ade selection medium were used to assess the transcriptional activation capability of EjMYB15.

### 2.6. Electrophoretic Mobility Shift Assay (EMSA)

The GST-tag recombinant EjMYB15 protein was constructed and purified using established protocols [31]. Wild-type (WT) or mutant probes containing MYB binding sites of target gene promoters were synthesized and 3′-end labeled with biotin by Sangon Biotech (Shanghai, China). The EMSA was performed using the Light Shift Chemiluminescent EMSA Kit (Beyotime Biotechnology, Nanjing, China) and detected by a ChemiDoc™ MP Imaging System (Bio-Rad, USA) using the chemiluminescence method [32].

### 2.7. Dual-Luciferase Report (DLR) Assay

For the evaluation of the transactivation activity of EjMYB15, the coding regions of *EjMYB15* were ligated to the pGreenII BD-62SK vector as effector. The reporter vector contained LUC driven by the 5 × GAL4 binding element and REN driven by the 35 S promoter. For the determination of the EjMYB15 activation on target genes, the CDS of *EjMYB15* was combined into the pGreenII 62-SK vector as effector, and the promoters of *EjCAT1*, *EjCAT2*, *EjGST1,* and *EjGST2* were subcloned into pGreenII 0800-LUC as reporter vectors. The recombinant constructs had been transiently expressed in tobacco leaves, and the LUC and REN activities were determined after 48 h [30]. The LUC/REN ratio reflects the transcription activity of EjMYB15 and its effect on the activation to target gene promoters.

### 2.8. Statistical Analysis

All experiments were performed in three replicates using a completely randomized design. Data were analyzed using SPSS 22.0. Differences between means were evaluated by Student’s *t*-test, with statistical significance set at * *p* < 0.05 and ** *p* < 0.01.

## 3. Results and Discussion

### 3.1. Melatonin Treatment Alleviates CI in Loquat Fruits

**Figure 1:**
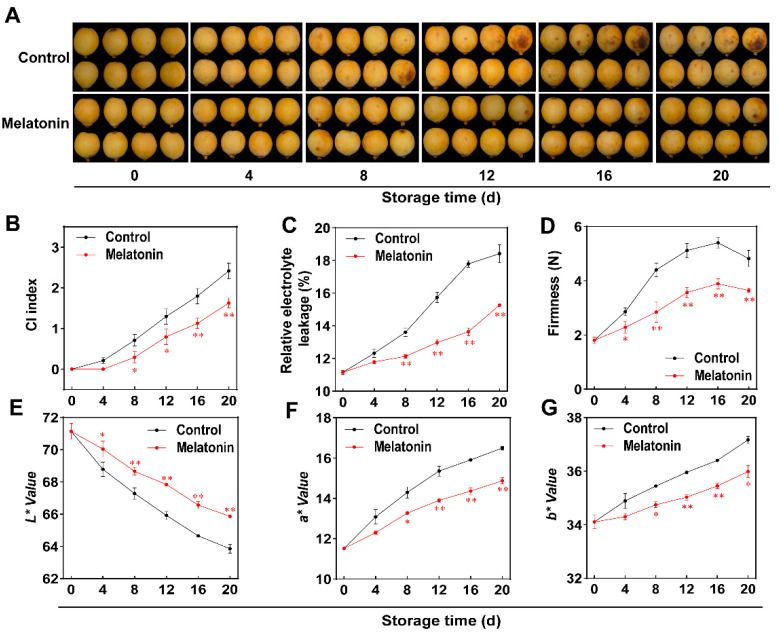
Melatonin treatment suppresses CI symptoms in loquat fruits. (**A**) CI symptoms in non-treated and MT-treated loquats during storage. (**B**) Change in CI index in MT-treated fruits. (**C**) Relative electrolyte leakage. (**D**) Fruit firmness. (**E**) Lightness (L*). (**F**) a* value. (**G**). b* value. Results are shown as mean ± SD (*n* = 3). Asterisks represent statistical significance (* *p* < 0.05; ** *p* < 0.01).

Chilling injury (CI) development is a critical factor driving quality deterioration and commercial value loss in postharvest cold-sensitive fruits [33]. As shown in Figure 1A, CI symptoms in loquats were evaluated during cold storage. The control loquats began exhibiting significant CI symptoms on day 8. Furthermore, with prolonged storage, the CI symptoms progressively worsened, manifested as a continuous expansion of the browning area on the fruit surface. In contrast, MT-treated fruits showed significantly alleviated CI symptoms (Figure 1A). CI index increased in both the non-treated and MT-treated groups during storage, but the melatonin-treated fruits had significantly lower levels than the non-treated fruits (Figure 1B). Similarly, relative electrolyte leakage increased in all samples during storage, while MT-treated loquats sustained lower values than the control. By the last day of storage (day 20), MT-treated loquats showed a 17.1% reduction in relative electrolyte leakage compared with the controls (Figure 1C). Fruit firmness in both experimental groups rose steadily over the first 16 days of storage, then decreased during the subsequent 4-day period (days 16–20). Specifically, on day 16, the control group exhibited a 0.384-fold higher firmness than the MT-treated group, with the latter maintaining consistently lower firmness values across the entire storage duration (Figure 1D). The L* value (lightness) of MT-treated fruits showed a continuous decline during the storage period but remained higher than that of the control group. By day 20, the L* value had decreased by 10.2% and 7.4% in control and treated fruits, respectively, compared with day 0 (Figure 1E). Both a* (redness) and b* (yellowness) values showed progressive increases in both control and MT-treated groups during storage, though the MT treatment significantly delayed these elevations compared to the control group (Figure 1F,G). The L* value represents the brightness of the fruit, with a higher L* value indicating a brighter appearance. The a* and b* values positively correspond to the red–green and yellow–blue color dimensions of the fruit, respectively. A higher a* value denotes a redder hue, while a higher b* value indicates a yellower hue. With the extension of storage duration, the symptoms of CI intensify progressively, characterized by the continuous enlargement of browned regions on the loquat surface. These browned regions have a heightened capacity for light absorption, thereby making the entire fruit appear visually lackluster. During CI development in loquat fruit, L* value decreases due to accumulated browning products (from oxidative stress) and damaged cell structure, both of which reduce light reflection. In contrast, the a* and b* values increase because chlorophyll degrades, making carotenoids (yellow-red pigments) more prominent, and browning intermediates also contribute to enhanced red and yellow hues. These results demonstrate that MT treatment effectively suppresses CI development in loquat fruits. Similar results were found in previous studies regarding peach [9], tomato [10], pear [11], plum [34], carambola [35], and banana [12]. Our findings further confirm that MT helps in alleviating the occurrence of CI in loquats under cold stress.

### 3.2. Molecular Characterization of EjMYB15

**Figure 2:**
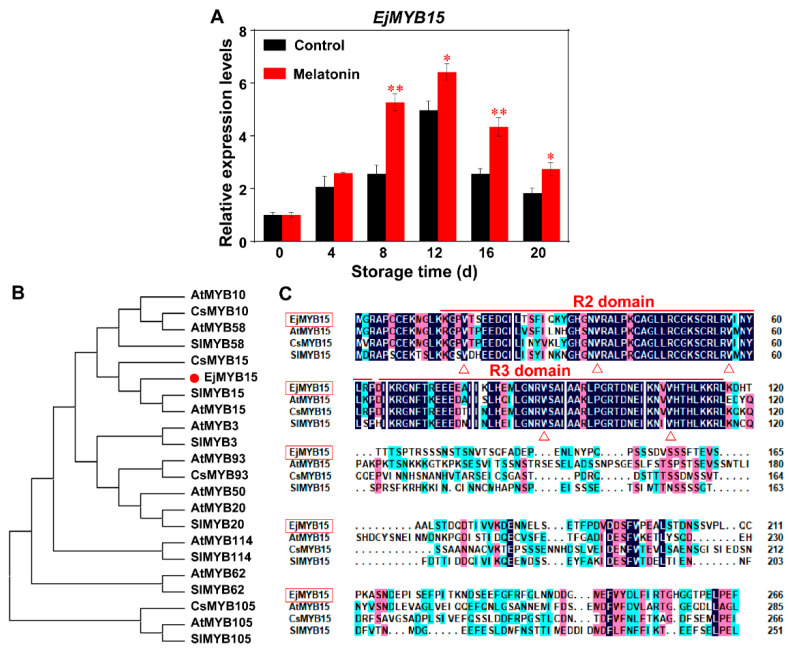
Identification of EjMYB15. (**A**) Expression profiles of *EjMYB15* in untreated and melatonin-treated loquats during cold storage. (**B**) Phylogenetic relationships of EjMYB15 with the MYB R2R3 subfamily members from Arabidopsis, citrus and tomato. (**C**) Multiple alignments of EjMYB15, CsMYB15, AtMYB15, and SlMYB15. Asterisks represent statistical significance (* *p* < 0.05; ** *p* < 0.01).

MYB proteins have been widely reported to respond to abiotic stress [19,20,21]. According to the conservation of MYB TF family in plant stress resistance pathways, we have identified a MYB TF, designated MjMYB15, from our transcriptome. As shown in Figure 2A, MT treatment enhanced the expression of *EjMYB15* during cold storage. Compared to the non-treated fruits, MT treatment led to a significant and sustained upregulation of *EjMYB15* expression in loquats throughout cold storage. At the peak point (day 12 of storage), *EjMYB15* expression in the MT-treated loquats was 1.292-fold higher than that of the control fruits. A phylogenetic tree was constructed to analyze the evolutionary connection between EjMYB15 and its homologous proteins from other plants. The results of comparisons show that EjMYB15 shares a high degree of homology with citrus CsMYB15, tomato SlMYB15, and Arabidopsis AtMYB15 (Figure 2B). Previous works have demonstrated that the CsMYB15 can respond to cold stress [32], and SlMYB15 positively regulates cold tolerance in tomato [36], implying EjMYB15 is associated with cold stress response. Multiple sequence alignment confirmed that EjMYB15 belongs to the R2R3-MYB TF family (Figure 2C) and shows significant potential for DNA binding.

**Figure 3:**
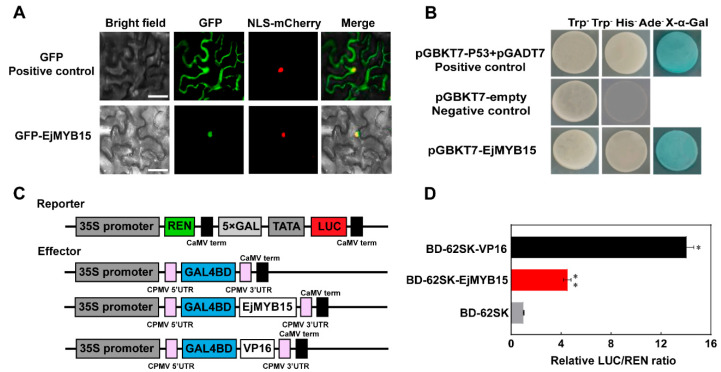
Molecular characterization of EjMYB15. (**A**) Nuclear localization of the EjMYB15 in *N. benthamiana* leaves. NLS-mCherry was co-transfected as a nuclear marker in each sample. Scale bar = 50 μm. (**B**) Transcriptional activity assay of EjMYB15 via yeast systems. (**C**) Schematics of the effector and reporter vectors employed for the dual-luciferase assay. (**D**) DLR assay of EjMYB15 in *N. benthamiana* leaves. Data are shown as mean ± SD (n = 3). Asterisks represent statistical significance (* *p* < 0.05; ** *p* < 0.01).

The EjMYB15-GFP fusion protein showed specific nuclear localization, as evidenced by its colocalization with the red fluorescent signal of the nuclear marker NLS-mCherry. In contrast, the GFP empty vector (used as a control) was dispersed throughout the entire cell. This demonstrates that EjMYB15 is a nuclear-localized protein (Figure 3A). The transactivation activity of EjMYB15 was further investigated using Y2H assay and DLR assays. As shown in Figure 3B, pGBKT7-p53+pGADT7 (the positive control) and pGBKT7-EjMYB15 transformants grew vigorously on the selective medium (SD/-Trp-His-Ade) and turned blue in the X-α-galactosidase assay, while pGBKT7-empty (the negative control) showed no growth or color change. EjMYB15 also exhibited transactivation activity in tobacco leaves. The LUC/REN ratio driven by BD-62SK-EjMYB15 was 4.55-fold higher than that of BD-62SK empty vector (the negative control) (Figure 3C,D). These results show EjMYB15 acts as a nuclear-localized transcriptional activator.

Previous studies have reported that several members of the MYB-R2R3 subfamily are key regulators of fruit responses to cold stress. For instance, the expression of PuMYB21 and PuMYB54 responds to cold stress during ‘Nanguo’ pear peel browning. Both PuMYB21 and PuMYB54 process the transcriptional activation activity [19]. MaMYB13 (one R2R3 MYB TF) processes transcriptional activation activity and is induced by low temperature, and is involved in response to cold stress in banana fruit [20]. Similarly, CaMYB340 is associated with CI occurrence in postharvest bell pepper [37], AhMYB30 increases freezing stress tolerance [21], and SlMYB13 is involved in cold resistance of tomatoes. These MYBs belong to the R2R3 MYB subfamily, which contains similar structural domains as EjMYB15 (Figure 2), further suggesting the potential important role of EjMYB15 associatied with CI development of loquat fruits.

### 3.3. Melatonin Treatment Induces the Expression of Antioxidant Enzyme Genes

**Figure 4:**
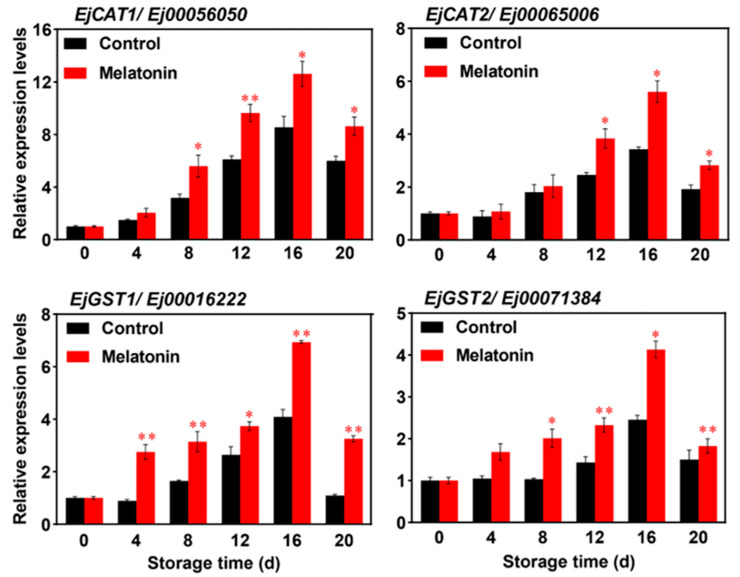
The expression level of antioxidant enzyme genes, including *EjCAT1*, *EjCAT2*, *EjGST1*, and *EjGST2* in untreated and melatonin-treated loquats during cold storage. Data are shown as mean ± SD (n = 3). Asterisks represent statistical significance (* *p* < 0.05; ** *p* < 0.01).

Our previous research demonstrated that MT alleviates CI development in loquat fruits by modulating ROS metabolism, particularly by significantly increasing the activities of enzymatic antioxidants and the contents of non-enzymatic antioxidants [8], but the molecular regulation is still unknown. Here, the possible involvement of antioxidant enzyme genes in MT-induced CI alleviation was investigated, and the expression of these genes in the control and MT-treated groups was detected by RT-qPCR. As shown in Figure 4, the expression levels of *EjCAT1*, *EjCAT2*, *EjGST1*, and *EjGST2* exhibited a dynamic trend of first increasing and then decreasing in both control and the MT-treatment groups during storage. However, the transcript levels of these four genes (*EjCAT1*, *EjCAT2*, *EjGST1*, and *EjGST2*) were markedly upregulated in MT-treated loquats compared to the controls. The gene expression levels in both groups peaked on day 16 of storage, followed by a gradual decline. At the peak, the expression levels of *EjCAT1*, *EjCAT2*, *EjGST1*, and *EjGST2* in the MT-treatment group were 0.475-, 0.633-, 0.697-, and 0.686-fold higher than those in the control group, respectively. Collectively, the present study confirms that MT induces the expression of *EjCAT1*, *EjCAT2*, *EjGST1*, and *EjGST2*, which is consistent with the MT-increased antioxidant enzymes activity and the antioxidant compounds accumulation in postharvest loquat fruits [8].

Previous studies have confirmed that the antioxidant defense system plays a crucial role in enhancing the cold resistance of various horticultural products [6,8]. Moreover, improving antioxidant capacity is widely recognized as an effective strategy to alleviate cold stress-induced oxidative damage in loquat fruits. For instance, CaCl_2_ treatment alleviated fruit CI by scavenging excessive ROS accumulation through enhancing antioxidant enzyme activities, related gene expression, and AsA-GSH cycle system [6]. Similarly, H_2_S treatment maintains ROS homeostasis by regulating the antioxidant system, thereby reducing oxidative stress during cold storage and effectively alleviating CI in loquat fruits [38]. Additionally, pre-treatment with *p*-coumaric acid (*p*-CA) reduces CI and preserves the postharvest quality of loquat fruits by improving the antioxidant syntheses and enhancing antioxidant enzyme activities [39]. Most recently, citrus essential oil (CEO) was found to alleviate browning in cold-treated loquat fruits through enhancing the antioxidant system via γ-aminobutyric acid (GABA)-mediated ROS metabolism [40]. Building on these findings, our findings further demonstrate that MT treatment alleviates CI development via increasing the expression of *EjCAT1*, *EjCAT2*, *EjGST1*, and *EjGST2* (Figure 4).

### 3.4. EjMYB15 Enhances the Transcriptions of Antioxidant Enzyme Genes

**Figure 5:**
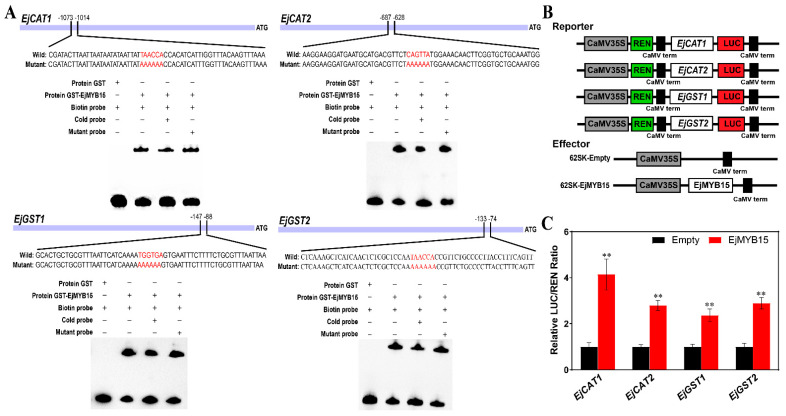
The binding and activation of EjMYB15 on the promoters of *EjCAT1*, *EjCAT2*, *EjGST1,* and *EjGST2*. (**A**) EMSA of EjMYB15 binds to the promoter sequences with MYB *cis*-elements from *EjCAT1*, *EjCAT2*, *EjGST1*, and *EjGST2*. These elements are indicated in red font. Cold probe and mutant probe were used as competitors. Shifted bands indicate the formation of DNA-protein complexes. The symbols − and + represent absence or presence, respectively. (**B**) Diagram of the reporter and effector vectors used in this assay. (**C**) EjMYB15 activated the transcription of *EjCAT1*, *EjCAT2*, *EjGST1,* and *EjGST2*, respectively. Data are shown as mean ± SD (n = 3). Asterisks represent statistical significance (** *p* < 0.01).

Promoter sequence analysis revealed that the promoter regions of *EjCAT1*, *EjCAT2*, *EjGST1*, and *EjGST2* contain the MYB cis-elements ACC(A/T)(A/C/T)(A/C/T) (Text S1). Therefore, EMSA was performed to verify whether EjMYB15 binds to these MYB cis-elements in the promoters of *EjCAT1*, *EjCAT2*, *EjGST1*, and *EjGST2*. The recombinant GST-EjMYB15 protein was successfully expressed in *E. coli*, purified, and confirmed by SDS-PAGE (Figure S1). Probes specifically containing the MYB cis-elements, isolated from the promoters of the four target genes (*EjCAT1*, *EjCAT2*, *EjGST1*, and *EjGST2*), were biotin-labeled, and unlabeled (cold) probes and mutated probes were used as competitors. As shown in Figure 5A, a mobility shift was shown when GST-EjMYB15 was incubated with the promoters of *EjCAT1*, *EjCAT2*, *EjGST1*, and *EjGST2*. However, the binding capacity of EjMYB15 to these promoters was significantly reduced in the presence of cold probes, whereas the addition of the mutated probes showed no significant effect. This demonstrates the specific interaction between EjMYB15 and the promoters of the four target genes.

Furthermore, DLR assay was performed on tobacco leaves to determine whether EjMYB15 transcriptionally activates *EjCAT1*, *EjCAT2*, *EjGST1*, and *EjGST2.* Reporter constructs were generated by individually fusing the LUC gene to the promoters of the four target genes. For the effector construct, EjMYB15 was driven by the 35S promoter. As displayed in Figure 5B, co-transfection with EjMYB15 significantly enhanced the transcriptional activities of *EjCAT1*, *EjCAT2*, *EjGST1*, and *EjGST2*. Compared to the negative control (empty 62SK vector), EjMYB15 remarkably increased the LUC/REN ratios for *EjCAT1*, *EjCAT2*, *EjGST1*, and *EjGST2* by 4.14-, 2.79-, 2.36- and 2.88-fold, respectively (Figure 5C). These results demonstrate that EjMYB15 activates the transcription of *EjCAT1*, *EjCAT2*, *EjGST1*, and *EjGST2* by directly targeting the MYB *cis*-elements in their promoters.

**Figure 6:**
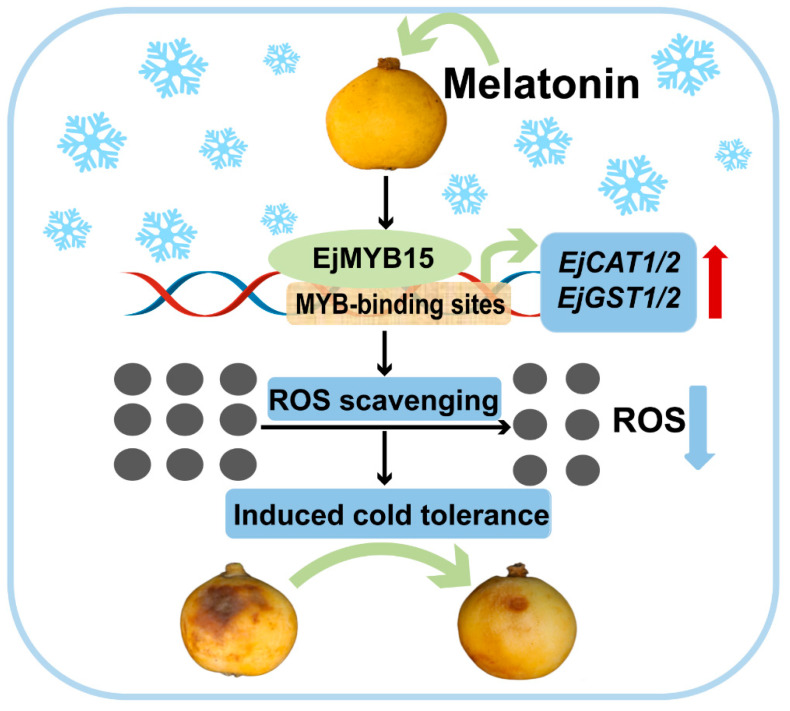
A working model of EjMYB15 in cold tolerance induction by activating ROS scavenging gene metabolism in postharvest loquat fruits. ROS are represented by gray circles.

The regulatory role of MYBs in ROS scavenging systems under cold stress has been extensively demonstrated. For instance, MdMYB23 promotes proanthocyanidin accumulation and enhances ROS scavenging capacity by binding to the promoter of *MdANR* (a key gene in proanthocyanidin synthesis) and activating its expression, thereby increasing the cold tolerance of apple fruit [41]. In chrysanthemum, DgMYB2 directly targets *DgGPX1*, significantly enhancing GPX enzyme to mitigate oxidative stress and thus improving freezing resistance [42]. Li et al. [43] overexpressed the *FvMYB82* in Arabidopsis and found that this gene effectively maintains the integrity of the lipid bilayer and ROS equilibrium under abiotic stress by increasing the activity of the antioxidant enzyme system, thereby significantly improving abiotic stress tolerance in plant. Similarly, Yao et al. [44] reported that overexpressing *MbMYB108* in Arabidopsis specifically increases the activities of CAT and POD, enhancing ROS scavenging capacity, which ultimately improves the plant’s tolerance to cold stress. Those findings are similar with our present study, in which EjMYB15 directly targets the MYB *cis*-elements in the promoters of *EjCAT1*, *EjCAT2*, *EjGST1*, and *EjGST2*, enhancing their transcription and ultimately improving cold tolerance of loquat fruits during storage. A working model illustrating the mechanism by which EjMYB15 mediates MT-alleviated CI in loquat fruits is proposed (Figure 6).

## 4. Conclusions

In summary, our findings clearly demonstrate that EjMYB15 mediates MT-induced cold tolerance in postharvest loquat fruits by transcriptionally activating antioxidant enzyme genes to enhance ROS scavenging capacity. MT treatment effectively alleviates CI in loquats during cold storage by enhancing antioxidant capacity and scavenging excessive ROS. Mechanistically, MT upregulates the transcript levels of *EjMYB15* and antioxidant enzyme genes, including *EjCAT1*, *EjCAT2*, *EjGST1*, and *EjGST2*. EjMYB15 is a nuclear-localized transcriptional activator that targets the promoters of these ROS scavenging genes, promoting their expression to reinforce antioxidant defense. This regulatory cascade ultimately enhances ROS scavenging efficiency and improves the cold tolerance of loquats. These results provide novel insights into the molecular regulatory network underlying MT-mediated CI mitigation and extend the theoretical basis for the application of MT in preserving cold-sensitive fruits.

## Data Availability

The original contributions presented in the study are included in the article/Supplementary Materials. Further inquiries can be directed to the corresponding authors.

## Supplementary Materials

The following supporting information can be downloaded at https://www.mdpi.com/article/10.3390/foods15020301/s1, Figure S1: SDS-PAGE gel of GST-EjMYB15 protein; Table S1. Summary of primers used in this study; Text S1: The cis-elements of MYB TFs in the nucleotide sequences of EjCAT1, EjCAT2, EjGST1 and EjGST2 promoters.
