# Supplementary material for: EjMYB15 Improves Cold Tolerance of Postharvest Loquat Fruit via Upregulating Antioxidant Enzyme Genes
Source: Foods. 2026 Jan 14;15(2):301. doi: 10.3390/foods15020301 (PMC12840339; doi:10.3390/foods15020301)

Supplementary Table S1. Summary of primers used in this study. GenBank accession numbers: *Ej00040204*（*EjMYB15*）, *Ej00056050*（*EjCAT1*）, *Ej00065006*（*EjCAT2*）, *Ej00016222*（*EjGST1*）, *Ej00071384*（*EjGST2*）GenBank no. JN004223 (*EjACT*).

Assay	Primer sequence (5'-3')	Restriction Site
**Full length cloning**	*EjMYB15-F:* ATGGGGAGAGCTCCTTGCTGT	
	EjMYB15-R: TCAAAATTCTGGTAATTCTGGCGTTC	
**Subcellular localization**	*EjMYB15- GFP -F:* ATCTAGAGCAGTCGACGGTACCATGGGGAGAGCTCCTTGCTGT	*Bam*H I
	EjMYB15- GFP -R: CTCCTCGCCCTTGCTCACCATAAATTCTGGTAATTCTGGCGTTC	*Kpn* I
**RT-qPCR**	*EjACT-qF:* AATGGAACTGGAATGGTCAAGGC	
	EjACT-qR: TGCCAGATCTTCTCCATGTCATCCCA	
	EjMYB15-qF: ATGGGGAGAGCTCCTTGCTGT	
	EjMYB15-qR: TCAAAATTCTGGTAATTCTGGCGTTC	
	EjCAT1-qF: CTGGGAGTTCCACAAGATTAC	
	EjCAT1-qR: GCTCCTCCAACCTTAATAGC	
	EjCAT2-qF: ACTCGACTCTTCGCCTATT	
	EjCAT2-qR: AGTAGTCAACCTCCTCATCTC	
	EjGST1-qF: AGGTTTATGCAGCCGTTAG	
	EjGST1-qR: GCATCTAGGAATGCTGGTATC	
	EjGST2-qF: CTCATCAATCTCAGCGACAA	
	EjGST2-qR: CAATGATCCCAACAATCACATC	
**Promoter isolation**	*EjCAT1 pro-F:* TGGTGACTGAAGCAATGTAGGC	
	EjCAT1 pro-R: GTAAGGATGCATGGACGAAGGACAG	
	EjCAT2 pro-F: CTCTCTATCTCCTCCAATCCGCT	
	EjCAT2 pro-R: GTAGGGATCCATGGAGGAAGGTC	
	EjGST1 pro-F: ATCTCAGAGACCAAAGTGAGGAG	
	EjGST1 pro-R: TTGTTAGGGTTATTGTTATCTATCAAATC	
	EjGST2 pro-F: TTGCCGATGTATGTGTCTCTG	
	EjGST2 pro-R: TAGAGAGCAGAGGGGGTAG	
**Y2H assay**	*EjMYB15-pGBKT7-F:* CATGGAGGCCGAATTCATGGGGAGAGCTCCTTGCTGT	*Eco*R I
	EjMYB15-pGBKT7-R: GCCGCTGCAGGTCGACGAAATTCTGGTAATTCTGGCGTTC	*Sal* I
**EMSA assay**	*EjMYB15-pGEX-F:* GGTTCCGCGTGGATCCATGGGGAGAGCTCCTTGCTGT	*Bam*H I
	EjMYB15-pGEX-R: AGTCACGATGCGGCCGCTCAAAATTCTGGTAATTCTGGCGTTC	*Not* I

	EjCAT1-probe-F: CGATACTTAATTAATAATATAATTATTAACCACCACATCATTTGGTTTACAAGTTTAAA<br>*EjCAT1-probe-R:* TTTAAACTTGTAAACCAAATGATGTGGTGGTTAATAATTATATTATTAATTAAGTATCG<br>*EjCAT2-probe-F:* AAGGAAGGATGAATGCATGACGTTCTCAGTTATGGAAACAACTTCGGTGCTGCAAATGG<br>*EjCAT2-probe-R:* CCATTTGCAGCACCGAAGTTGTTTCCATAACTGAGAACGTCATGCATTCATCCTTCCTT<br>*EjGST1-probe-F:* GCACTGCTGCGTTTAATTCATCAAAATGGTGAGTGAATTTCTTTTCTGCGTTTAATTAA<br>*EjGST1-probe-R:* TTAATTAAACGCAGAAAAGAAATTCACTCACCATTTTGATGAATTAAACGCAGCAGTGC<br>*EjGST2-probe-F:* CTCAAAGCTCATCAACTCTCGCTCCATAACCAACCGTTCTGCCCCGTTACCTTTCAGTT<br>*EjGST2-probe-R:* AACTGAAAGGTAACGGGGCAGAACGGTTGGTTATGGAGCGAGAGTTGATGAGCTTTGAG	
**Dual-luciferase transient expression assay**	*EjMYB15-BD-62SK-F:* CGCCGTCTAGAACTAGTGGATCCATGGGGAGAGCTCCTTGCTGT	*Bam*H I
	EjMYB15-BD-62SK-R: TCGATAAGCTTGATATCGAATTCTCAAAATTCTGGTAATTCTGGCGTTC	*Eco*R I
	EjMYB15-62SK-F: GGCCGCTCTAGAACTAGTGGATCCATGGGGAGAGCTCCTTGCTGT	*Bam*H I
	EjMYB15-62SK-R: ATCGATAAGCTTGATATCGAATTAAATTCTGGTAATTCTGGCGTTC	*Eco*R I
	EjCAT1 pro-LUC-F: TATAGGGCGAATTGGGTACCTGGTGACTGAAGCAATGTAGGC	*Bam*H I
	EjCAT1 pro-LUC-R: TTGGCGTCTTCCATGGGTAAGGATGCATGGACGAAGGACAG	*Hind* III
	EjCAT2 pro-LUC-F: TATAGGGCGAATTGGGTACCCTCTCTATCTCCTCCAATCCGCT	*Bam*H I
	EjCAT2 pro-LUC-R: TTGGCGTCTTCCATGGGTAGGGATCCATGGAGGAAGGTC	*Hind* III
	EjGST1 pro-LUC-F: TATAGGGCGAATTGGGTACCATCTCAGAGACCAAAGTGAGGAG	*Bam*H I
	EjGST1 pro-LUC-R: TTGGCGTCTTCCATGGGTAAGGATGCATGGACGAAGGA	*Hind* III
	EjGST2 pro-LUC-F: TATAGGGCGAATTGGGTACCTTGCCGATGTATGTGTCTCTG	*Bam*H I
	EjGST2 pro-LUC-R: TTGGCGTCTTCCATGGTAGAGAGCAGAGGGGGTAG	*Hind* III

Supplementary Text S1. The *cis*-elements of MYB TFs in the nucleotide sequences of *EjCAT1*、*EjCAT2*、*EjGST1* and *EjGST2* promoters. *cis*-elements of MYB TFs were underlined. The probe nucleotide sequence used in EMSA assay is indicated in bold. Translation start site (ATG) was shown in yellow.

>*EjCAT1* （Ej00056050）

TGGTGACTGAAGCAATGTAGGCTAGATAGTTTGGACTTGTGGGCTCGTGGCATAAGATTGTTGTAATGGATGACACCATCAATGTTTAA
AAATATAATTCTAAGATAGAAAGAAAGCTAAAAATGTAGAAACCCACGTAACTTAGGCACGTGATAGACTATAAGTGATAGAAGAGGA
AAATGATGTACATGCAAGAGGATTTTTCCATGCATCATTTTTAGCATTTTGTTATAATTAATCATTAAATTGAATAAAGCAATAAAAATCCT
CATATATATTTATCATTCGAGAAAGGGCAATGTTAAGGAAATCAAAATTTTAAACTAAATTTGCAAACTTAGTTTACAAGTATTAAGTAAG
TGTCAATTAATTTGCAAACTTAATTTTCAAATTATATTATCTTGAGAAAGGGCAACGTATAATTAATAATACGTTAAT**CGATACTTAATTA**
ATAATATAATTATTAACCACCACATCATTTGGTTTACAAGTTTAAAAATTTGGTCCCTAGCATTACCCTTTGAGAAATGTCAAGACTC
TCTCAAATGTCGGATTCTTCACTCTCTGACACCTCATGTTTTTGAAACAATATTGTATAATGTTGAAACGAGAATTAACATTAAATTATAA
GTTGGGAGAGAGTGCATGAAGAATCATATTTGAGAGAATCTCCTTAAGCATTTCTCCATTCATTTTTGTACATAAATGCAACAAAATTTC
GTTAAAAAAAATTGGAGATAATCATTCCAACTAGTGACTGAGTATTTATCATTTATAGGCAAAATTTAACCTCTAAATTCATAAATGTCAG
TTTTTACAAAATCTTTCAAATGTAATAAAAAAATATAATTGAATTGACCTAAATACATACAAAATTAAAACCCATGTTATTTGGGATTCTC
ACCCGCTATCCCTCCTTCATCTTCTTCCTCCATACAAAAAACCAATTTTCAAGCCGAAGCCCCATTTCAACGACTTTAAACATACACTCA
AAAACCTAATTTGGTTTCTTCCTATATTGAGAGGTAGCCCGATGGGAACTTTCATCCTTAGGGTTACAGATTTTTCAAAAATAATATTATT
TTATTGATATTGGACCAAACCACCACTAAGATTCCGTGCACCAAAAATTGAAATTGAATAATAAAATAGCAATAAAATTAAAGAAATTAG
AGCATGAAAATAAGGAAACCAAGTTGAACCATACCAATCAAATGGAAAAAGCGAATTTTAACCCAAGAAAAAAAAAAAAAAAACTTAA
AACTTAATTACAAAAAACTGACACCAGTATTACCAACTCGTAACAATATGAAACCAAAAAAATTATATATTTTAATCCTCTTTTAATTGAT
ATTCAATTAACTCGAGTATTTACCGGTGAGAGCTTGTCTATTTTCATTGTGTGGAACTCTCATGTCTATAATTCTCTCCTCTTCGATTGTCA
TTTGAGTGAGTGGCTCTCCTCTTACCTTCTCCATCAACCCCTCTCTCACTCTAACCTGTCCTTCGTCCATG

>*EjCAT2* (Ej00065006)

CTCTCTATCTCCTCCAATCCGCTCAATGTTTTCTATTTCCTCCAATCATTTCTTACTTTCTTTCCTTCTCTCTCCTACTTTCCTTCATCCTCT
CTCCTCTTTCTCTCTTGACCTCCTCTTTTTGGATCTCTTTATCCAGTTTAAGTTGTAAGATTCAAAACTTTTAAATCGCACACCAACAAGT
TTTTGAGTCTTCAAGAAAATTGTTTTCAAGAAAAATTCTTAAGAAATATTTTGAGAAATTATAAAAATTTTCAAATACAATATCAAACAA
GCCTTAAGAGAATTTGAGTATTTTAATATTAACAAACCCAATCTACTTGTTTCATAAAAAAAAAAAAACAAAAAACAAAAAACTCCAGTA
GTTTTATTATTTTTTTAATATTTAACAGGTGAATATTGCTTCCTAAATTTCAAAAATGATTGGTCCGTTGCTATAATTAAATAGTCTTTTGCA
ACATTTATTAGAGATAAATTTTTTAAATTTAATTTTTTTTAACAAACGATATTATCTATATTAAAAGAGAGAAGATGGGTTTAGCCTCATAA
TAATAAGAATCGAGTCTAAGATCTCTCACTTATACATGAGAAGGAATACCACTGGACAGTAATATTATGTGGCTAAATTTAACTTGTTAAA
ATAGTATAAGCTTTTCAGTGCGTCGAGAACACGGTTCAATCTACTAGTGTCATAATATTATTAATTGATAGAAAATTTATCTCATAATGTAA
TGATACTTGGTGCACAAAACCATGTTTCTGACCTATTAAAAATCTCTTCTAAGAGTTTTGATTGTTTAATATAGAGTAAGTCACCCGGCAT
CAGTGGTTATGCATAGATGTTAGCAAAGCACCAAAACTAGGAT**AAGGAAGGATGAATGCATGACGTTCTCAGTTATGGAAACAACT**
TCGGTGCTGCAAATGGCCTAAAATACACGGACACGTGTTTGACTTTTGATTAAAAAATTTAAAAATTAGATATGTGTTAGCGTAATTGG
TTATTAGCAACTGCTGATTTTTCAACAGCAAAATCTTTTTCCTCAATTATTCACATTGATTTTTGCTACTGCTGATCTTTCAATAGCGAAAT
CTGGTTCCTCTCAATTATTCACATTGATTTTTGATACTGCTGATCTGTCAACGGCGAAGTCTGGTTCCTCAATTATTCACATTGATTTTTTC
CACCTCGCTCTCTGTCTTCTTGCGATCACCATACAAAATATCAGCAAGCAAAGTGGCAAGTTGGCAAGTTGGCGATAATCATTCCGAGC
GGAAGACATGCAGTGACCTTCCAGCTGTGTCTGTGGATAAAGATAACGTGGGGCCGAAAGCGGAAAATCGTAATTTTACACAACAGCG
CGTGGGACGCCCACGACATATCCAAAGCCAAAACTCAGCGTTTATAAATTTGCTTGCATTTCAGCCTTCGATTGCCCATCTGAGCGAGT
GGCTTACCCCTTCCTCTGTTCACCTCTTACCTTCTACGTCAAGCTCTCTCTCTCTCTCTCTCTAACCTGACCTTCCTCCATG

>*EjGST1* (Ej00016222)

TGCATAACTCCTTATTTAGTTCTTGAGATTTGAAATAGATAGAATTTGTCTTTGAGTTTGTCCACAATCAATTATTTTGATCATTCCATGA
AAAATCTCCATAAAATAAAAATAAAATGACAAAAAAAAAATACCCTCAATTTTGGTCAAATCATTTTGGCATATTGTTTATTAAATTGAGG
GTAATTTTGTCATTTTGATCATTATTTAACATAATTTATTACCCAAGGACTAAAATGATTGATGATGGACAATCTCAAGAACCACTTCTATT
GATTTCAAATCTCAGAGACCAAAGTGAGGAGTTATGCAAATCTCAGAAACCATTTTAACTAAAAAGCCGTTTTGAATATAATCTAAGTT
TGAAAAATTAACGGATGTGACATTTGTAATATGTTAGACACTTATTTAGAATATTTAAAGGGATTTGGATCCTCTCTATTTGCTCAAGAGA

GGATCCAAATCTTATTTAAAGAGATGTGATATCCACACACCTCATTTTACTTCTCACACATCTTTTTAATTTTCGGCTGTCGGATCTGATG
AATTGAAGAAGATCAACTGATAAAAAATATCAAGATGTGTGTGAGAGAAGATCAACTGATAAAAAATATCAAGATGTGTGTGAGAAGT
AAAATGAAATATGGATAGCACATCCCTATTTAAAAAAGTTGAAACCAATTTGAATCAACACTAAAATTTTTAAGGTTTTAAGTACTTAAT
CCACACACACACACACACACACACACACAAAGCGCAAACGGCGAAAAACTATAGTAAGTCAAACAAAGTAATAGAATAAAGGGCCATTT
TAAAATAGGTCCAAAAATACTAAAAATTGGACCTATTTCAAAAGTAGGGTTATAAAATTAGACTTATTTCAAATTTTCCCAAAAATTAAA
TTGAGGTTCAACTTAATCCTCGCCTTGGTATCTGTCTGTCAGAACTCAAAACGTAATAGAATAAGGACATGGATTGTCTCCTCCTAGTTT
TGGTGCCCTCTCATGCACTTCTATTTGTGTGGTCACGGTTAAATCACGTCAGTATTTTATATTATTTTTTTATAGAGATAATAAGACAAAAA
ATAAACAGAAGGGCATGAGAGGGCACCGAAAATAGGAGGGCAGACAATCCATATCCATAGAATAATCTGTCTGTCAAACCTCAAAAGC
TGAAAACTCAGGGCCGGCTGAAGCTGATGCT**GCACTGCTGCGTTTAATTCATCAAAATGGTGAGTGAATTTCTTTTCTGCGTTTA
ATTAA**TATTCGGTTGATTGATTTGATTTGATAGATAACAATAACCCTAACAAATG

>*EjGST2* (Ej00071384)

CCATATTTGCCACTTGATTGAGCCTCACGTGCGTGACACGTGACTCATTTAGACAAATTCTGACAGAGTTGATGAAAGGAACCATAGAT
GCATGTTTTAAGGGACCAATAATTGTAGATTTTTAGTTGAAGAACCATTGCTCCAATTCAATTAAAGATGATCGACCATTGTTACAATTTT
CTCTGAATGATAAATCAACTAACCACATATCACATACCAATTCAAAGTCAGAGGCTTTTGAATTTGTTACGTTTCAATTCTCTTTAATTTT
CTTGTAACTTAATTTGTCTTTGAACACACACAAAAAAATAAAAATAAAAAATTAATTGACTGATTTTCTTTGTAAACGCAGTTTTTGCCGA
TGTATGTGTCTCTGAAAATTGGTTAAGTGCATTGTAAATGATGTAATTAACACTAACATGCTATTAAAATAATGAAAACTATTTCTACTTTA
TTAAACAAATAAAATTAATGTCTTCTTTGAGGAAATTCCAACAGAGAAACCATTTCTTTTCGAGAAGATGTTATCTTTATTTTCCTTGTTA
AATGATGTTGTATTTGTTTTATTACTGGTGGTTTTTCAGTTAATATATATATATATTCATTATAGTTAAATGATGTTAATATTTGTTGATCTGG
AAAATTCTAGTCTTTTTAATTACTTTTATTTCGGACAAATGATCTACCAATTTTTATGGTCTTATTATTAATTGGTTTGCACTATGAAATTAT
CATTTAGTCTCATTTTATTTTTTAAAACTAGTTTTGTCTTACTTCACCTCTTGAAATTGTCGATTTCATATTATTTAATCTCAATTCATTTTAT
TTTCATTTATTCCATCAAATAAATATTAAATTAAAATGTACTATGAACATTTTATTTTTGACATGGGAAAAATAAGTCTTACACTTCGTGAC
ATGACACTTACGAGCTACAAGGATGAACTAATATTAAATTAGCATTTGTCTACACACTTTGTATATATGAACACATTTTTTTAGAAAATGA
GAAGGAAAGAGAGAGGGAGAGAGAGAACATGGAGGATTCGGGAGGTTTTTGTTTTATTTATTTATTATTTTAAATATTAAAGATATTTTA
ACATTACTTGTAGGTGAGGTTTCAATAAAAAAATAGTAAAATTTAGACATGTGAATTTATATTATTGCCCATCATTTTTTGTGTGATAGAAG
ATTAAGTTATCTTTTCACCTGCTTTTGATTAACAAATAGGATTTCATTAATTAATAAAGATGAGACGAAAAGATAATTTTAAGAAACAAAA
TGTGAGTAAATAAATTTCTATGGTTAAAATATGATTGAATACAATTTTAGAGAAAAATTTATTAATAAGCCTTTTATATGCAAAGTGTATTA
AGTTTGTGTCAGCACTGACCTAATATATATATATATATATATATATATATATATATATATGC**CTCAAAGCTCATCAACTCTCGCTCCATAACCA
ACCGTTCTGCCCCGTTACCTTTCAGTT**TCATCTTCCACACTCTCTACCCCCTCTGCTCTCTAATG

Supplementary Figure S1. SDS-PAGE gel demonstrating purification of the GST-tag EjMYB15.

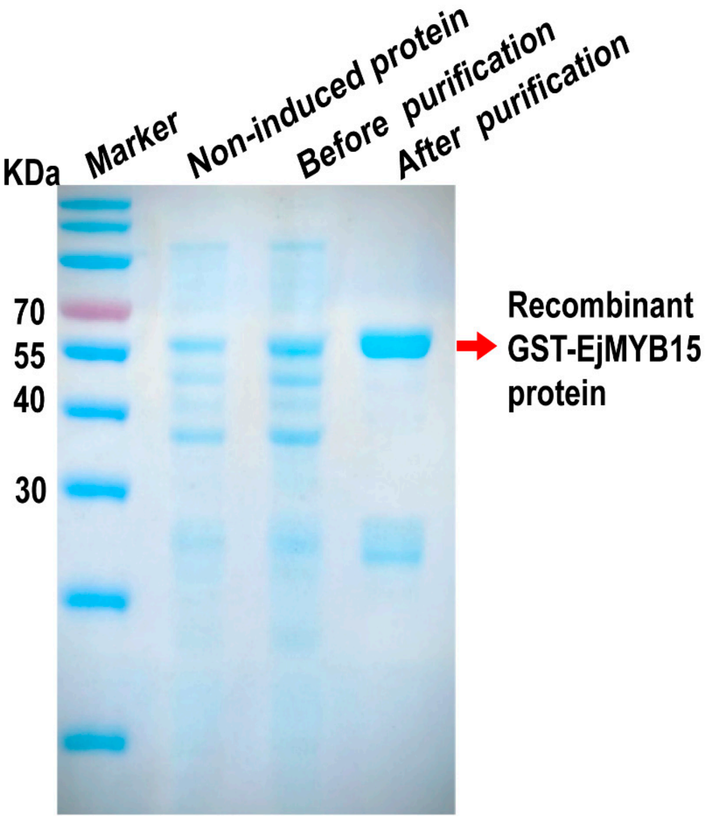

Supplement: Supplementary file 1 [file foods-15-00301-s001.zip › foods-4070845-supplementary.pdf]
